# Supplementary material for: Artificial Intelligence to Predict the BRAF V595E Mutation in Canine Urinary Bladder Urothelial Carcinomas
Source: Animals (Basel). 2023 Jul 25;13(15):2404. doi: 10.3390/ani13152404 (PMC10416820; doi:10.3390/ani13152404)
Supplement: Supplementary file 1 [file animals-13-02404-s001.zip › animals-2473732-supplementary.pdf]

Table S1: Comparison of AI-based BRAF mutation prediction with confirmed PCR result based on sample quality and specific histomorphological features.

	**PCR**			
**AI prediction (all samples)**				
	Positive	Negative	Total	
Positive	40	10	50	
Negative	29	17	46	
Total	69	27	96	SE 58%, SP 63%
**AI prediction (high quality samples)**				
Positive	24	4	28	
Negative	3	3	6	
Total	27	7	34	SE 89%, SP 43%
**AI prediction (standard quality samples)**				
Positive	7	1	8	
Negative	8	3	11	
Total	15	4	19	SE 47%, SP 75%
**AI prediction (poor quality samples)**				
Positive	9	5	14	
Negative	18	11	29	
Total	27	16	43	SE 33%, SP 69%
**AI prediction (classic urothelial only)**				
Positive	34	8	42	
Negative	6	2	8	
Total	40	10	50	SE 85%, SP 20%
**AI prediction (non classic urothelial only)**				
Positive	6	2	8	
Negative	23	15	38	
Total	29	17	46	SE 21%, SP 88%
**AI prediction (invasive only)**				
Positive	8	2	10	
Negative	15	9	24	
Total	23	11	34	SE 35%, SP 82%
**AI prediction (non-invasive)**				
Positive	16	3	19	
Negative	3	1	4	
Total	19	4	23	SE 84%, SP 25%
**AI prediction (inflammatory)**				
Positive	8	3	11	
Negative	20	8	28	
Total	28	11	39	
**AI prediction (non-inflammatory)**				SE 29%, SP, 73%
Positive	32	7	39	
Negative	8	9	17	
Total	40	16	56	

Table S2: Extension of Table 4. Assessing the AI tool reliability for correct *BRAF* mutation prediction based on specific features across different sample cohorts. A Chi-square test was performed for categorical data (urothelial differentiation, inflammation, slide quality, invasive growth, and mutation status) and a 2-sided t-test for continuous data (sample size). For each feature, p values are indicated for PCR positive and negative cases, as well as separately for true positive and true negative prediction. P values of <0.05 were considered significant (in bold).

**Feature**	**BRAF PCR**	**All samples**	**Standard and high quality samples only**	**High quality samples only**
Urothelial differentiation	Pos and neg	**<0.01**	>0.1	>0.9
	Pos	**<0.00001**	**<0.001**	>0.19
	Neg	**<0.001**	**<0.006**	**<0.009**
Inflammation	Pos and neg	**<0.01**	**<0.03**	>0.7
	Pos	**<0.00001**	**<0.0005**	>0.48
	Neg	>0.38	>0.15	NA
Slide quality	Pos and neg	**<0.02**	NA	NA
	Pos	**<0.0001**		
	Neg	>0.42		
Sample size (ROI)	Pos and neg	**<0.02**	>0.08	>0.3
	Pos	**<0.003**	>0.09	>0.2
	Neg	>0.3	>0.5	>0.9
Invasive growth	Pos and neg	>0.08	>0.3	>0.2
	Pos	**<0.002**	>0.23	>0.28
	Neg	**<0.04**	>0.57	>0.68
Mutation status (PCR)	NA	>0.6	>0.1	**<0.02**
